# Anæsthetics in Hospital Practice

**Published:** 1860-09

**Authors:** 


### *Anæsthetics in Hospital Practice.*

The discovery of anæsthetics was universally hailed as a great and unqualified blessing to man, the victim of unavoidable pain. The year of the announcement of the power of ether to render the patient insensible under the hand of the operator, was distinguished as the *annus mirabilis;* it began a new era in the history of operative surgery, and the older surgeon, in the language of the elder Warren, "wished again to go through his career under the new auspices." We can well imagine with what enthusiasm he who had been accustomed to struggle through difficult operations on patients forcibly held, now pursued his dissections as on the cadaver, and saw the patient, on the completion of the operation, suddenly restored to a full possession of all his faculties, as if by magic. And when, a year or more after these first experiments with ether, chloroform was introduced to notice, so agreeable to the senses, so prompt in its action, and so harmless in its effects, the perfection of anææsthetic agencies was thought to have been attained.

But every good must have its corresponding ill. It was soon announced that a lady, sitting in a dentist's chair, had suddenly expired while inhaling chloroform preparatory to the extraction of a tooth. A second, third, and fourth case was reported, and always previously to some trivial operation. The faith of its friends, however, remained unshaken, and these unfortunate results were attributed to the attending circumstances, and not to the anæsthetic. At length fatal cases began to occur occasionally in hospitals, in the presence of eminent physicians and surgeons, and in spite of their previous precautions, and efforts to resuscitate the victim. Finally, the fact seemed established beyond peradventure, that chloroform is not an innocuous agent, even under circumstances apparently the most favorable for its administration, by the occurrence of a fatal case (in a dentist's chair, however,) in spite of the persistent and well directed efforts of Professor Simpson himself to restore animation.

It can now no longer be denied that anæsthetics are followed by unpleasant and occasionally fatal effects in a given number of instances. The latest statistics that have been published are as follows:—Total fatal cases in Europe, one hundred and twentyfive. When we take into account the aggregate cases of anæsthetization during the last sixteen or seventeen years, of their almost universal use in hospitals, and in private practice, this mortality is a percentage of the whole number of cases, positively infinitesimal. It is doubtful if any active remedy of the materia medica can show a better record.

The recent death by chloroform in Bellevue Hospital has, we understand, raised the question in the Medical Board as to the propriety of allowing this agent to be longer employed for purposes of anæsthesia in that institution. Before this question can be properly decided, the comparative merits of ether and chloroform must be considered, for anæsthetics in some form are now indispensable to the practice of operative surgery and midwifery, and can never be discarded, even though the mortality from their use were tenfold its present per centage. And before chloroform is stricken from the list, it were well to inquire as to the real sources of danger from its use, for if it is demonstrated that under circumstances it is as safe as any anæsthetic, every surgeon will, under such circumstances, prefer chloroform.

The comparative merits of ether and chloroform, as anæsthetics, it is not easy to decide. The statistics which we have given above show, that of the one hundred and twenty-five fatal cases from anæsthetics in Europe, twenty-five occurred during the inhalation of ether, and one hundred of chloroform, giving a mortality from the latter equal to four-fifths of all the cases. Although chloroform would seem by this exhibit to be the more fatal anæsthetic, yet a moment's reflection will convince any one that it may not even approximate the truth, for we have no knowledge of the per-centage of the deaths to the number of cases of administration of either agent. It might, and probably would appear, could we sift this subject thoroughly, that chloroform had been given four times as often as ether during that period. We may, however, arrive at a very satisfactory conclusion as to the safety of chloroform, by taking the gross number of cases of its administration in certain well-authenticated instances, and noting the results. For example, it was given twenty-five thousand times by the French, in the Crimean war, without a single fatal issue. It is freely used in midwifery by many eminent English and American obstetricians, and, we believe, no fatal case has yet been reported in this department of practice. Professor Simpson is stated to have used from five to seven gallons annually for thirteen years, without an unfavorable result.

The real sources of danger in the employment of chloroform have not been sufficiently studied. Authors mention:—1st, *A full stomach;* for vomiting being a common symptom in chloroform inhalation, the patient is liable to be suffocated. 2d, Affections of the nervous system, as delirium tremens, epilepsy, hysteria, etc. 3d, Affections of the vascular system, as fatty degeneration of the heart, atheromateous deposits, etc., etc. We do not propose to discuss these contra-indications to the use of chloroform, as it is by no means as yet established how far these conditions are to be regarded as complicating its effects. We believe, however, that it was maintained by the late Dr. Snow, whose opinion on all subjects relating to chloroform is entitled to our confidence, that even when lesions of the nervous and vascular systems do exist, chloroform properly administered, is far less dangerous than an operation without an anææsthetic. From some recent investigations as to the nature of death from chloroform, the following interesting facts appear:—1st, That the great majority of deaths (two-thirds) occur in slight operations, as those performed on sphincters, tendinous sheaths, strabismus, tooth-drawing, etc., but few during the larger operations, as amputations, resections, ovariotomy, etc. 2d, In the majority of fatal cases by chloroform, death occurred before the operation,—during the first stage of inhalation—the stage of excitement. 3d, That the deaths that have occurred after the operation, and were attributable to the anæsthetic, have generally been when ether was slowly administered, or ether and chloroform, but not pure chloroform. Without dwelling on these subjects, which are all of the deepest interest to those who are discussing the question of the relative or actual merits of the different anæsthetics, we shall allude to what we consider, if not the real, certainly the great source of danger in the use of anæsthetics in general, and chloroform in particular, in our hospitals we refer to the gross and culpable carelessness of their administration. Rarely is the patient carefully examined by a competent person to determine if there be any contra-indication to the use of anæsthetics—a point that should never be neglected. The delicate and most responsible task of administering the agent is usually committed to a junior physician, who has no knowledge whatever of the nature of his duties; he knows nothing of the different stages through which the patient is to pass, or of the value of the symptoms which appear during the administration; his inhaler is a towel well saturated, and his directions often are to apply it directly to the face. The stage of *profound coma* having been reached, the operator seizes the scalpel, and all eyes are directed to its movements; the innocent junior, all absorbed in the operation, forgets his duty, unconsciously drops the towel upon the patient's face, and occasionally adds the weight of his body to its suffocating effect, as he leans forward in the anxious pursuit of knowledge. At length a moan, or the collapse of the jetting arteries, or the suggestion of a bystander more interested in the sufferer than the operation, recalls attention to the condition of the patient. Naturally enough he has ceased to breathe; the operation is suspended; the messenger is despatched for brandy; and in the meantime artificial respiration by the most improved method is attempted by every available means. Fortunately the patient is generally resuscitated, at least sufficiently to have the operation completed, and be taken to the ward.

We do not here give an overdrawn picture, for such scenes if haply not more unpleasant, may be witnessed in our hospitals almost weekly. The reform should commence with the mode of administration of these agents. A medical man of known ability should be selected to administer the anæsthetic; we say medical man, because he will not become so much interested in the operation as to forget his duties. To his care should be committed, so far as practicable, every patient who is about to submit to an operation. This is but that precaution which every surgeon exercises in private practice, and hence the few cases of death from anæsthetics which occur outside of our hospitals. If this degree of care is exercised in our hospitals and still fatal consequences follow the use of ether or chloroform, or both, the question may well be raised as to the propriety of rejecting the more dangerous.—*Am. Med. Times.*

